# Lipoprotein Metabolism, Protein Aggregation, and Alzheimer’s Disease: A Literature Review

**DOI:** 10.3390/ijms24032944

**Published:** 2023-02-02

**Authors:** Elena Grao-Cruces, Carmen M. Claro-Cala, Sergio Montserrat-de la Paz, Clevio Nobrega

**Affiliations:** 1 Department of Medical Biochemistry, Molecular Biology and Immunology, School of Medicine, University of Seville, 41009 Sevilla, Spain; 2 Department of Pharmacology, Pediatrics, and Radiology, School of Medicine, University of Seville, 41009 Sevilla, Spain; 3 ABC-RI—Algarve Biomedical Center Research Institute, Faculty of Medicine and Biomedical Sciences, University of Algarve, 8005-139 Faro, Portugal

**Keywords:** amyloid, apolipoprotein, lipoprotein, lipid metabolism, stress granules, tau

## Abstract

Alzheimer’s disease (AD) is the most common form of dementia. The physiopathology of AD is well described by the presence of two neuropathological features: amyloid plaques and tau neurofibrillary tangles. In the last decade, neuroinflammation and cellular stress have gained importance as key factors in the development and pathology of AD. Chronic cellular stress occurs in degenerating neurons. Stress Granules (SGs) are nonmembranous organelles formed as a response to stress, with a protective role; however, SGs have been noted to turn into pathological and neurotoxic features when stress is chronic, and they are related to an increased tau aggregation. On the other hand, correct lipid metabolism is essential to good function of the brain; apolipoproteins are highly associated with risk of AD, and impaired cholesterol efflux and lipid transport are associated with an increased risk of AD. In this review, we provide an insight into the relationship between cellular stress, SGs, protein aggregation, and lipid metabolism in AD.

## 1. Introduction

Alzheimer’s disease (AD) is the most common form of dementia; 60–70% of all dementia cases correspond to AD according to WHO. In the United States alone it is estimated to affect 6.2 million people, a number that is expected to increase rapidly in the coming years [1,2]. In Europe, the incidence of AD and other dementias is 188.01 (per 100,000 population). AD symptomatology is initially associated with impaired memory [3] and is defined by the presence of two neuropathological features: amyloid-beta (Aβ) plaques and tau neurofibrillary tangles (NFT) [4]. These neuropathological events are associated with progressive neuronal loss and chronic neuroinflammation in AD [5,6].

AD is classified as familiar and sporadic. The cause of familiar AD, commonly early-onset, is associated with three major mutations in genes related to amyloid-beta precursor protein (APP) processing, while the cause of sporadic AD, commonly late-onset, remain unknown. However, there are different risk factors for sporadic AD that include age, gender, apolipoprotein E (apoE) genotype and metabolic disorders such as diabetes, hypertension, and hypercholesterolemia, among others [7]. The apoE gene codifies a lipoprotein that works as the main lipid transporter in the brain. This gene has three different alleles ε2, ε3, and ε4 that result in apoE2, apoE3 and apoE4 isoforms; the apoE4 isoform is the main genetic risk factor for sporadic AD [8]. Homozygosis of ε4 increases the risk of AD 12-fold, while in heterozygosis, the risk is increased 3-fold [9]. However, the presence of ε4 is not a direct cause of AD developing.

Despite the wide knowledge of AD, the initiators in sporadic AD remain undefined. While the apparition of amyloid plaques and/or NFTs is well defined as AD pathology, other molecular processes, such as neuroinflammation, autophagy, and cellular stress, contribute to the development of AD and are believed to play a key role in the onset of AD. The immune response in the brain is mainly controlled by microglial cells [10]. Different factors produce pro-inflammatory activation of the microglia, such as protein aggregates. Abnormal Aβ accumulation and NFTs induce a pro-inflammatory environment in the brain, leading to neuroinflammatory responses [5,6]. Additionally, the pro-inflammatory environment is known to increase Aβ accumulation and NFTs [11], producing an endless loop of protein aggregation and neuroinflammation that ends in neuronal loss. Neuroinflammation and protein aggregation are not the only key factors in this endless loop; additionally, an imbalanced proteostasis usually occurs in neurodegenerative disorders, including AD. Malfunction in those protein degradation pathways increases protein aggregation and neuroinflammation, producing an overload of the protein-degrading processes [12].

The aim of this review is to provide an overview of the relationship between AD protein aggregates and lipid metabolism.

## 2. Protein Aggregation and AD

AD is pathophysiologically characterized by an accumulation of abnormal Aβ plaques and NFTs. Proteostasis, or protein homeostasis, is essential in neurons and even more so in the context of a neurodegenerative disease. Proteases and chaperones are essential proteins for the maintenance of proteostasis, in combination with the ubiquitin–proteasome system (UPS), the endosomal–lysosomal pathway, and the autophagy–lysosome system. The UPS, the autophagy system, and the endosomal–lysosomal pathway are the three pathways that degrade intracellular proteins [13].

Chaperones are multidomain proteins involved in protein folding, unfolding, and turnover. Heat Shock Protein 70 (HSP70) and 90 (HSP90) are two of the most abundant and ubiquitous chaperones. HSP70 is involved in protein folding, protein translocation among organelles and disassembly of aggregates [14]. On the other hand, HSP90 binds non-native peptides preventing their aggregation and intervenes in the late stages of protein folding [15]. The UPS is a protein degrading system in which proteins are ubiquitinated in order to be recognized by the proteasome and be degraded, however, the UPS has recently been discovered to have other cell signaling functions [16]. Autophagy is a process that clears dysfunctional organelles, long-lived proteins, protein aggregates, and pathogens. The endosomal–lysosomal pathway interferes in different steps with autophagy, is another degrading machinery of cells and involves the formation of endosomes [17]. In fact, neuron survival depends on the role of UPS, autophagy, and the endosomal–lysosomal pathway. Among neurodegenerative disorders, the accumulation of misfolded proteins is characteristic and impairments in protein degrading machinery are relevant factors in their development, causing synaptic dysfunction, alteration of axonal transport, and neuronal loss [18].

NFTs are fibrillary intracytoplasmic structures constituted of paired helical filaments (PHFs) of tau protein formed in neurons. Tau protein’s main function is to stabilize axonal microtubules, and tau phosphorylation and dephosphorylation are essential for correct functioning of axonal traffic [19]. However, hyperphosphorylation of tau induces a change in its conformation, producing a prion-like molecule that seeds tau self-assembly, leading to the formation of tau aggregates, and tau aggregates form twisted PHFs, which are the main constituents of NFTs [20]. The initiator of pathological tau phosphorylation remains unknown [21], but misfolded and aggregated hyperphosphorylated tau produces an overload in protein clearance pathways that produces an accumulation of hyperphosphorylated tau. For example, Tai et al. in 2012 found that tau and hyperphosphorylated tau tend to accumulate in the presynaptic axon and the postsynaptic soma, and hyperphosphorylated tau is also polyubiquitinated but resistant to degradation. Furthermore, the accumulation of hyperphosphorylated tau was associated with UPS dysfunction [22]. Additionally, hyperphosphorylated tau synaptic accumulation is associated with the accumulation of immature autophagic vacuoles (AVs) [23,24], which is the first sign of autophagy deficits, and could be due to enhanced autophagy induction and impaired or insufficient later lysosomal degradation [25]. In addition, the maturation of AVs depends on their transport through the neuron, therefore, if the microtubule system is impaired due to tau hyperphosphorylation in AD, it could enhance autophagy failure and explain AV accumulation [26]. Tau homeostasis is also controlled by the action of chaperones, for example, HSP70 induces tau binding to microtubules and tau correct function because it blocks tau aggregation. In 2018, Kundel et al. reported that HSP70 sequesters tau oligomers and neutralizes tau seeding and aggregation [27]. Additionally, phosphorylated tau binds to the HSP70/HSP90 machinery, blocking tau seeding and aggregation [28]. HSP90 forms complexes with late-folding tau and helps to correct tau folding [29], however, HSP90 does not bind phosphorylated tau alone, it needs to form a complex with HSP70 [28]. However, there are controversial results regarding HSP90 and its co-activators, because recent evidence suggests an association between HPS90 and tau pathology, specifically, HSP90 and its ATPase homolog increase tau phosphorylation [30,31,32].

Aβ plaques are extracellular microscopic lesions that have a core of Aβ peptide surrounded by enlarged axonal endings. The Aβ peptide is produced by the cleavage of a transmembrane protein known as APP. APP can follow two different cleavage pathways (amyloidogenic and nonamyloidogenic pathways), and the proteases that produce APP cleavage determine the product peptide. In the non-amyloidogenic pathway, APP is cleaved by α-secretase and produces sAPPα and C83 fragment. On the contrary, in the amyloidogenic pathway, sequential cleavage by β and γ secretase can produce Aβ peptides with 37–49 amino acids depending on the site of cleavage of γ-secretase. Aβ40 is the most abundant peptide, Aβ42 is less abundant but is the product peptide that has the greater tendency to aggregate [33]. Despite the fact that Aβ plaques are extracellular features, their formation is preceded by intracellular intermediates, such as monomeric isoforms. Therefore, protein degrading machinery is essential to degrade Aβ peptides and clear initial Aβ accumulation [34]. Recent evidence points to autophagy and the UPS as the protein degrading systems that clear Aβ42 oligomeric species, and to the endosomal–lysosomal pathway playing a key role in the degradation of monomeric Aβ42 [35]. The proteostasis imbalance that usually occurs in pathological conditions and ageing, produces the accumulation of APP metabolites, and autophagy failure impedes the secretion of Aβ [36]. On the other hand, part of the APP processing route is directed to endosomes and lysosomes for alternative degradation or for the generation of longer Aβ, creating an intracellular pool of pathologic Aβ [37]. Additionally, there are different LOAD risk factors, genes associated with the endosome-lysosome pathway, such as SORL1, PICALM, BIN1, and CD2AP, among others [38].

Amyloid fibrils have been described as disassembling because of HSP70-related proteins. In fact, α-synuclein fibrils disassemble by the binding of HSP70 and other related chaperones [39]. Although α-synuclein is not a main feature in AD, recent evidence has described α-synuclein fibrils degenerating neurons in AD [40]. This evidence surrounding amyloid fibrils and HSP70 could open a new question of the effects of HSP70 on the initial accumulation of Aβ peptides on amyloid fibrils.

The initiators of tau hyperphosphorylation and Aβ42 accumulation remain unclear. Different factors are known to influence protein aggregation. Traditionally, it has been suggested that Aβ pathology induces tau pathology [41,42,43,44]. This theory has been widely studied and discussed, mostly due to the paradox of spatial and temporal distribution of Aβ and tau pathology [45,46,47,48,49,50]. Some authors have described independent actions of Aβ and tau pathology, discarding the precedence of Aβ pathology to tau pathology [44,51,52]. However, recent studies focused on the synergy of Aβ and tau pathologies have shown that both pathological features could act in coordination and culminate in enhanced neurodegeneration. For example, Aβ amyloid has been reported to stabilize tau aggregates [53], and tau pathology induction has been proposed to produce amyloid accumulation in models with no previous amyloid deposits, and to enhance amyloid accumulation with pre-existing amyloid plaques [54]. Additionally, authors have reported that Aβ and tau pathology as a whole is more than the sum of the independent parts [55]. However, what is clear is that once protein accumulation has started, an overload of protein-degrading mechanisms occurs, and the process becomes an endless loop that produces chronic cellular stress and neuroinflammation. The process can also start backwards; prolonged neuroinflammation and cellular stress can induce proteostasis imbalance, leading to the accumulation of protein aggregates.

Proteostasis is also important to the correct maintenance of synapses, in healthy neurons and AD neurons. Coordinated protein synthesis and degradation are essential for all the modifications that occur in synapses, because synapses are highly energy-demanding regions with rapid protein turnover. Therefore, dysfunction in cellular degradation pathways, which occurs in AD, would induce synapsis malfunction and neurodegeneration. For example, Aβ accumulation mediates the ubiquitination of AMPA receptors and therefore it would induce AMPA receptor turnover, reducing neurotransmission [56]. On the other hand, Aβ accumulation increases the binding of glutamate to NMDA receptors. This leads to excitotoxicity, and would produce a pathological influx of the calcium in neurons [57], and calcium levels influence the UPS [58].

Chronic neuroinflammation is another key factor of AD, and it is reported to alter cellular proteostasis. During an inflammatory process, protein degradation is usually downregulated, the UPS system activity is reduced [59], and autophagy is activated as a compensatory mechanism that induces endoplasmic reticulum (ER) stress and consequently impairs endosomal–lysosomal degrading pathways [60]. These authors suggest that chronic neuroinflammation leads to impaired proteostasis, despite the compensatory mechanisms, due to a reduction of the UPS and the endosomal–lysosomal pathway as a result of ER stress [59,60]. Neuroinflammation could occur because of protein aggregation during the progress of AD, however, neuroinflammation is a common feature in ageing. It is reported that the microglia tend to a proinflammatory-prone phenotype, producing a chronic activation of the immune response and its consequences [61,62,63].

## 3. Stress Granules and AD Pathology

Stress granules (SGs) are nonmembranous aggregates formed as a response to stress stimuli. Their composition is variable, but the main components are translation factors, mRNAs, and RNA-binding proteins (RBPs). While approximately 50% of the SG proteins are RBPs, proteins in SGs that do not bind RNA include metabolic and post-translation modification enzymes, and protein and RNA remodeling complexes, and these proteins are recruited into SGs by protein–protein interactions [64]. SGs are formed as a cytoprotective mechanism against cellular stress. They are involved in the regulation of translation, mRNA storage and stabilization and cell signaling during stress [65].

SGs are very dynamic. They are assembled when different stress stimuli occur and disassembled after the stimuli is gone. However, pathological factors, such as imbalanced proteostasis, neuroinflammation, and chronic stress, lead to the deposition of pathological SGs. For the formation of SGs, RBPs and nontranslated RNA are attached during the nucleation phase of SG formation. The nucleation proteins are multidomain proteins that facilitate the assembly of SGs. SG formation continues to create a stable form of the SG core with RNA and RBPs. Later, different interactions with the SG core and other cell components create the primary biphasic core/shell and then larger mature SGs are formed in a process dependent on microtubules. When the stress stimuli is gone, SGs disassemble by the combined action of chaperones, microtubules, autophagy mechanisms, and post-translational modifications [66]. In different pathological conditions, such as AD, where autophagy is usually impaired and the microtubule system is usually less efficient due to tau hyperphosphorylation, assembly and disassembly is dysregulated and SGs become a pathological feature [67]. Therefore, the accumulation of pathological SGs and the associated dysfunction of the cytoprotective mechanisms are key factors in the development of neurodegenerative diseases. SGs have been reported as pathological features in other neurodegenerative diseases, including AD [68]. For example, in AD, SGs contain tau protein, indicating that pathology of AD is associated with SGs [69]. Furthermore, in neurodegenerative diseases, many SG-associated protein mutations and alterations in the assembly of SGs have been described, which could be leading factors in the accumulation of pathological SGs [68].

**Figure 1:**
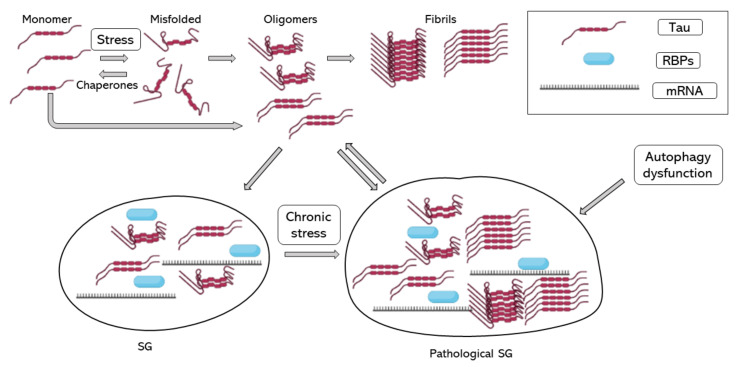
Tau aggregation and SG assembly. Tau folding–misfolding is controlled by chaperones, stress signals produce a disequilibrium and enhance tau misfolding. Tau misfolding and hyperphosphorylation induce tau aggregation into oligomers and fibrils. Tau oligomers induce tau binding to RBPs, such as TIA-1 and the formation of SG. When stress is chronic, and SGs do not disassemble, SGs turn into pathological SGs, which enhance tau aggregation. RBPs, RNA binding proteins; SG, stress granule.

In 2012, Vanderweyde et al. described the co-localization of hyperphosphorylated tau and SGs in two transgenic models of tau pathology and human cases of AD. In addition, they reported that co-localization of tau and SGs depended on the severity of the disease. They reported the co-localization of T-cell-restricted intracellular antigen-1 (TIA-1) and G3BP, two of the core RBPs recruited during SG formation [70]. Recent evidence shows that TIA-1 interacts with misfolded and aggregated tau, and as tau pathology worsens, TIA-1 accumulation increases in neurons [71,72,73,74]. Harris et al. in 2016 proposed that SGs contribute to tau misfolding, suggesting that tau plays an important role in neuronal RBP biology and that pathological SGs induce tau toxicity [72]. Maziuk et al. in 2018 developed a study of the tau interactome which described and confirmed tau interaction and co-localization with different RBPs [73]. Additionally, Ash et al. in 2021 demonstrated that TIA-1 is sufficient to drive tau self-assembly at physiological protein concentrations, unlike other core SG RBPs, such as G3BP, which also interact with tau but in the presence of crowding agents [71]. Other studies of the tau interactome found that the tau interactome is dysregulated during disease [75] and found a relationship between RNA metilation and tau-containing SGs through the RBP HNRNPA2B1 [76]. A study of the RNA-binding proteome in rapidly progressive AD identified that splicing factor proline and glutamine rich (SFPQ) is down-regulated and dislocated from the nucleus to co-localize with TIA-1. SFPQ was found to co-localize with pathological tau, suggesting that SFPQ could play a role in tau hyperphosphorilation. Additionally, in vitro studies showed that SFPQ is recruited in TIA-1 SGs and co-localizes with tau [77]. One of the possible links between tau pathology and SG accumulation could be mTOR activation during chronic stress; the inhibition of mTOR is essential to autophagy activation and the impaired autophagy is associated with higher tau aggregates and SGs. On the other hand, the good function of the cytoskeleton is essential for autophagy function and SG assembly and disassembly, though the increase in tau phosphorylation and aggregation by failure of autophagy activation would only increase autophagy failure and SG accumulation, increasing stress stimuli [78]. Taken together, SG formation could induce tau pathology, and pathological accumulation of SG would contribute to AD progression (Figure 1).

Although most of the research regarding SGs and AD is focused on tau pathology, Aβ pathology is also related to SG formation. Aβ deposits seed the aggregation of TDP-43, which is a nuclear protein that is translocated into the cytosol to form SGs. Therefore, Aβ pathology induces the aggregation of SG associated proteins [79]. On the other hand, TDP-43 worsens AD pathology by binding to Aβ oligomers and forming toxic complexes [80]. Furthermore, it was found that Aβ plaques and tau were found to co-accumulate with TDP-43 [81,82].

Other features of AD could contribute to the formation of pathological SGs, by inducing chronic cellular stress or activating different kinases. For example, AD-associated protein aggregates could activate SG formation through activation of the unfolded protein response (UPR) [83], which can occur in response to cellular oxidative damage in AD [84]. Impaired lipid metabolism in AD, associated with apoE genotypes and/or lipoprotein dysfunction, has been reported to induce chronic cellular stress [85], contributing to pathological SG formation. Additionally, the apoE genotype itself has also been reported to influence SG formation. In an organoid derived from iPSC, cells from an AD patient with the ApoE4 genotype presented accelerated SG formation, which was described as a consequence of an overexpression of the core SG RBPs [86]. On the other hand, it has been reported that SG dynamism is lost with aging [87,88]. This could be the result of aging-impaired proteostasis derived from chronic neuroinflammation. Ageing is a key factor of LOAD, therefore the normal ageing process would lead to cell stress and pathologic accumulation of SGs.

## 4. Protein Aggregation and Apolipoprotein Variants in AD Pathology

Most of the research regarding protein aggregation and apolipoproteins is focused on apoE. Numerous studies have shown the role of ApoE peptides in neurodegeneration and AD. ApoE is differentially cleaved depending on the isoform, and the toxicity of apoE peptides is also isoform-dependent. ApoE4 has more susceptibility to proteolytic cleavage than apoE3 and apoE2, and its peptides are the most neurotoxic. Different proteases mediate apoE cleavage and the protein structure determines the susceptibility to fragmentation [89,90]. ApoE is cleaved in the C-terminal domain in neurons, and this fragment binds to Aβ and localizes in amyloid plaques, whereas the lipidated form of ApoE, in the N-terminal domain interacts with NFTs.

Different authors have shown that apoE can impede cellular clearance of Aβ, and the underlying mechanism between Aβ and apoE was proposed to be competition, as heparin-binding proteins, for cell surface heparin sulphate proteo-glycans (HSPG). The HSPG pathway was previously suggested to be a common route for the cellular uptake of tau and α-syn [91], hence in theory apoE may also be able to also alter the cellular uptake of both of these molecules. In particular, apoE4 has been reported to promote Aβ fibrillization directly, but it may also contribute to AD pathogenesis via Aβ-independent mechanisms including tau phosphorylation.

On the other hand, the accumulation of tau protein in neurons is promoted by the imbalance between the functions of tau protein kinases and phosphatases. Consequently, C-terminal truncated ApoE fragments in the neuron have been identified with raised tau phosphorylation suggesting that they may play a key role in AD-associated neuronal deficits [90]. Another study showed a direct association between the apoE4 isoform and tau hyperphosphorylation by activation of GSK-3β leading to subsequent tau pathology [92] Consequently, C-terminal truncated ApoE fragments induce oxidative stress and inflammation, releasing pro-inflammatory cytokines. Increased matrix metallopeptidase 9 (MMP-9) leads to β-site amyloid precursor protein–cleaving enzyme-1 (BACE-1) activation and/or apoptosis, and interleukin (IL)-1β interacting with neurons via LDL-related protein receptor (LRP) induces NFT formation [93].

ApoE genotype has been reported to influence the mitochondrial and ER stress response. The mitochondria and the reticulum are highly associated with reactive oxygen species (ROS) production and are connected organelles; therefore, dysfunction could easily pass from one to the other. The ER is a major protein folding compartment, and authors suggest that apoE4 could be recognized as unfolded by the ER, and that apoE4 blocks ER vesicle traffic. This would provoke an ER stress response and then cause the activation of the UPR. The main action is to stop translation, in order to restore protein homeostasis. However, progressive and chronic activation of UPR by apoE4 would lead to cell dysfunction, high production of ROS and cellular stress [94]. Additionally, neuron specific apoE4 C-terminal fragments cause mitochondria dysfunction by triggering ER stress [95]. ER and mitochondrial malfunction and high ROS production would activate the SG response, and therefore chronic stress stimuli would lead to chronic and pathological SGs. On the other hand, apoE4 and apoE3 C-terminal fragments, especially those of apoE4, interact with tau and induce NFT production in neurons [96].

## 5. Brain Lipid Metabolism and AD

The connection between AD and lipid metabolism is supported by multiple risk factors for late-onset AD that include lipid metabolism-related genes, such as apoE, apoJ, PICALM, ATP-binding cassette A1 (ABCA1) and A7 (ABCA7), and low-density lipoprotein (LDL) receptor protein 1 (LRP1). In addition, there is the need for correct lipid homeostasis for good cell functioning.

Lipid metabolism in the brain is the combination of complex and highly coordinated processes regarding glial cells and neurons. Neurons have a low fatty acid degradation capacity, so glial cells, such as astrocytes and microglia, clear and degrade the excess fatty acid in neurons. On the other hand, cholesterol is an essential lipid in neurons, because it is essential for synaptogenesis, and its primarily direction of transport is from glial cells to neurons. In fact, alterations in brain cholesterol metabolism have been shown to be related to AD and other neurodegenerative diseases. Growing research shows that the brain metabolic lipid is modified with ageing by accumulation of lipid droplets. Histological staining has shown that older mice had more lipid droplets within microglia in the hippocampus compared to younger mice, and significantly larger in size [97]. In a mouse model, tau aggregation correlated with a lower expression of CYP46A1, a crucial enzyme for cholesterol synthesis in the brain, and CYP46A1 deficits were strongly associated with severe memory deficits [98,99,100]. Therefore, lipid transport is a key factor for proper function of the brain, and alterations could result in neuronal loss of function.

Lipid transport is mainly controlled by lipoproteins, lipoproteins do not usually cross the blood–brain barrier (BBB), so the lipoproteins circulating in the brain are usually formed in the brain, where the main apolipoproteins are apoE and apoJ. Certain apoE genotypes [8] and apoJ SNPs are described as AD risk factors [101], due to the reduced function of these apolipoproteins and the consequent malfunction of lipid transport in the brain.

High-density lipoprotein-like particles in the brain are formed by binding phospholipids and cholesterol to apoE. ApoE-containing lipoproteins are the main cholesterol transporters in the brain, and the flux is mainly from astrocytes to neurons. ApoE interacts with different receptors in neurons, including LRP1 and ABCA1. Therefore, reduced function of any of these proteins would lead to impaired lipid transport. For example, apoE-deficient mice have been demonstrated to develop age-associated cognitive deficits [102], LRP1 knockout is associated with neuroinflammation and synapse loss [103], and the lack of a copy of ABCA1 exacerbates memory deficits associated with the apoe4 allele [104]. However, in human late-onset AD, there is not a mutation/genotype that produces total depletion of function; usually, there is a reduced or impaired function. For example, the apoE4 genotype produces a reduced capacity to binding, secreting, and transporting lipids [105]. Additionally, apoE is a molecule that contributes to Aβ clearance because it can bind to Aβ peptides [106]. A reduced function of apoE therefore not only produces an imbalance in lipid metabolism, but also a reduction in Aβ clearance, both processes contributing to the progress of AD.

Lipid flux from neurons to glial cells is essential when a pro-inflammatory stimuli occurs and during neuroinflammation, because neurons would not clear peroxidized lipids [107]. Therefore, during AD, when neuroinflammation is chronic and oxidative damage is continuous, defective lipid transport would only increase oxidative damage accumulation in neurons and would cause neuronal death. Additionally, lipoproteins interact with glial cell receptors, such as triggering receptor expressed in myeloid cells 2 (TREM2). TREM2 is related to anti-inflammatory signaling in microglia. As with Aβ clearance and loss of function, mutations in the TREM2 gene are defined as causing AD risk. TREM2 has different ligands, including LDLs, apoE, and lipidated apoJ. Therefore, not only mutations in TREM2 would lead to an impairment of its function, but also mutations in its ligands [108].

Brain lipid composition is known to change during ageing in healthy humans and is associated with a compensatory anti-inflammatory response [109]. The change in the lipid content of the brain is associated with neuronal loss of function [110]. On the other hand, brains with AD show a different lipid pattern; for example, an increase in pro-inflammatory lipid mediators has been found in human CSF and post mortem brains with AD [111].

## 6. Apolipoproteins and AD

### 6.1. ApoE

ApoE is the most studied apolipoprotein in relation to AD (Table 1) because the apoE genotype is the main risk factor for late-onset AD. Astrocytes are the main producers of apoE, however, neurons also express the apoE gene and could contribute to apoE brain levels [112]. ApoE is found not only in HDL-like particles, but also in VLDLs and LDLs, and the apoE genotype defines the tendency to form one or another type of lipoprotein. For example, while apoE3 and apoE2 tend to form small HDLs rich in phospholipids, apoE4 associates preferentially with large VLDL particles [113]. ApoE-containing lipoproteins interact with different cell surface receptors, such as LDLR, LRP, VLDLR and ApoE receptor 2 [114]. However, the apoE isoform would determine the ability to bind to different receptors, for example, apoE2 shows lower capacity to bind to LDLR, which is associated with the protective role of apoE2 genotype against AD [115,116].

ApoE isoforms also differ in susceptibility to proteolytic cleavage; apoE4 is more susceptible than apoE3 [89]. Additionally, the proteolytic result of apoE4 cleavage was found to cause neurotoxicity: for example, apoE4 fragments have been reported to form NFT-like aggregates or damage the mitochondria [90]. Additionally, the apoE isoform has been reported to influence BBB integrity. ApoE4 has been reported to increase MMP-9 resulting in the degradation of tight junctions and the reduced coverage of the astrocyte end foot of blood vessels [117,118].

On the other hand, the apoE isoform is also associated with neuroinflammation, in specific, the apoE4 genotype is associated with proinflammatory responses that contribute to AD development. However, apoE4 influence on inflammation is not a direct consequence of the genotype, is due to impaired lipid metabolism [119].

### 6.2. ApoJ

ApoJ or clusterin is a heterodimeric protein (Table 1), which is highly expressed in the brain. ApoJ forms HDL-like particles in the brain, despite the fact that it has a lower tendency to lipidate compared to apoE and is essential to maintain lipid homeostasis in the brain. Initially, apoJ was described as a chaperone that reduces misfolded protein aggregation [120]. Additionally, evidence describes different roles of apoJ in Aβ accumulation [121]. There are two types of apoJ, intracellular and extracellular apoJ, and both have different functions. Intracellular apoJ is involved in stress response regulation [122], however, intracellular apoJ could act as a neurotoxic factor, impairing DNA repair, and is known to interact with Tau in AD patients [123]. On the other hand, extracellular apoJ is known to bind Aβ peptide and increase its clearance, acting as a neuroprotective factor [123]. Extracellular apoJ can bind to different cell surface receptors, including TREM2. Aβ peptide is obtained and degraded by microglia better when it is complexed with apolipoproteins, such as apoJ [108].

Different apoJ polymorphisms are associated with a higher risk of AD [124,125], however, the molecular mechanisms underlying the risks associated with each polymorphism are poorly studied.

### 6.3. ApoCI

ApoCI is a small apolipoprotein contained in HDLs codified by the APOCI gene (Table 1), which is in an expression cluster with APOE, APOCII and APOCIV [126]. Polymorphisms in the APOCI gene and its promotor are associated with increased risk of AD [127]. ApoCI is a direct modulator of lipoprotein metabolism, mainly by promoting HDL maturation via LCAT activation [128]. Additionally, ApoCI induces the expression of a transcriptional hepatic repressor that reduces the expression of different lipid metabolism genes and lipoprotein assembly [129]. In the brain, ApoCI is synthesized by astrocytes, but with a lower expression and secretion rate than apoE [130].

The relationship between APOCI polymorphisms and AD remains unclear. Some authors even suggest that apoCI is associated with AD through the effects of the polymorphisms on apoE expression, due to its co-localization with the APOE gene in a cluster [127]. However, other studies have showed that AD patients have lower apoCI mRNA levels but higher apoCI protein levels [130] and that apoCI co-localizes with Aβ plaques [131]. On the other hand, Cudaback et al. [132] reported that apoCI modulates microglia activation, with an inverse relationship between apoCI concentration and pro-inflammatory cytokines.

## 7. Conclusions

**Figure 2:**
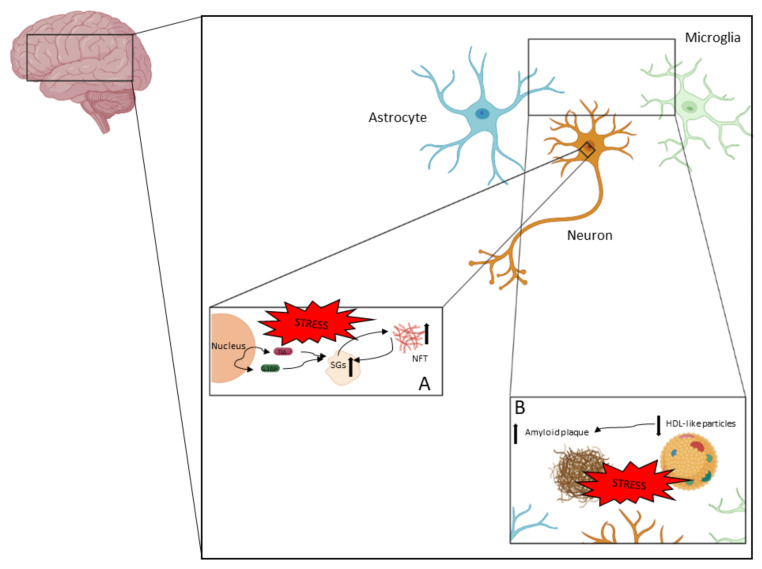
AD is a neurodegenerative disease in which neurodegeneration is driven by different processes. NFTs and amyloid plaques are the two main pathological features in AD. (**A**) NFTs are accumulations of hyperphosphorylated tau and other components. Tau hyperphosphorylation and aggregation could be driven by the pathological accumulation of SGs, which could be a consequence of chronic stress. (**B**) Amyloid plaque is an extracellular pathological feature (with intracellular intermediates) that mainly contains Aβ peptides. Amyloid plaque formation is highly associated with the clearance capacity of apolipoproteins contained in HDL-like particles.

AD is a complex neurodegenerative disease in which different processes take place. In Figure 2, the pathophysiological aspects described in this review are represented.

The main conclusions are:

- Alzheimer’s disease is a complex disease with an important inflammatory factor;
- The formation of SGs during cellular stress could be a protective mechanism against cell death;
- Pathological SG formation due to chronic stress would increase tau hyperphosphorylation and aggregation;
- Lipid metabolism plays an essential role in AD development;
- Apolipoproteins are key factors for AD development; not only the apoE genotype plays a role in AD.

## Figures and Tables

**Table 1:** Apolipoprotein function and relationship with AD.

Apolipoprotein		Function	AD
	Variants		
ApoE	ApoE2	Cholesterol transport from glial cells to neurons, Aβ clearance	Protective
	ApoE3		Neutral
	ApoE4		Risk factor
ApoJ	Intracellular	Stress response regulation	Could act as neurotoxic
	Extracellular	Lipid transport, Aβ clearance	Polymorphisms with loss of function, risk factor
ApoCI		Modulator of lipoprotein remodeling	Unclear, different polymorphisms are described as risk factors

## Data Availability

Not applicable.
